# Sterols from *Mytilidae* Show Anti-Aging and Neuroprotective Effects via Anti-Oxidative Activity

**DOI:** 10.3390/ijms151221660

**Published:** 2014-11-25

**Authors:** Yujuan Sun, Yanfei Lin, Xueli Cao, Lan Xiang, Jianhua Qi

**Affiliations:** College of Pharmaceutical Sciences, Zhejiang University, Yu Hang Tang Road 866, Hangzhou 310058, China; E-Mails: 11119014@zju.edu.cn (Y.S.); 11219008@zju.edu.cn (Y.L.); 11319006@zju.edu.cn (X.C.)

**Keywords:** neuroprotection, anti-aging, anti-oxidative stress, *Mytilidae*, sterols, *UTH1*, *SOD*, Alzheimer’s disease

## Abstract

For screening anti-aging samples from marine natural products, K6001 yeast strain was employed as a bioassay system. The active mussel extract was separated to give an active sterol fraction (SF). SF was further purified, and four sterol compounds were obtained. Their structures were determined to be cholesterol (CHOL), brassicasterol, crinosterol, and 24-methylenecholesterol. All compounds showed similar anti-aging activity. To understand the action mechanism involved, anti-oxidative experiments, reactive oxygen species (ROS) assays, and malondialdehyde (MDA) tests were performed on the most abundant compound, CHOL. Results indicated that treatment with CHOL increases the survival rate of yeast under oxidative stress and decreases ROS and MDA levels. In addition, mutations of *uth1*, *skn7*, *sod1*, and *sod2*, which feature a K6001 background, were employed and the lifespans of the mutations were not affected by CHOL. These results demonstrate that CHOL exerts anti-aging effects via anti-oxidative stress. Based on the connection between neuroprotection and anti-aging, neuroprotective experiments were performed in PC12 cells. Paraquat was used to induce oxidative stress and the results showed that the CHOL and SF protect the PC12 cells from the injury induced by paraquat. In addition, these substance exhibited nerve growth factor (NGF) mimic activities again confirmed their neuroprotective function.

## 1. Introduction

With improvement of material life and medical conditions, the lifespan of human becomes longer and longer. Meanwhile, we face the biggest problem which age-related diseases like Alzheimer disease (AD) are endlessly increased. Aging is an important factor of the pathogenesis of age-related diseases [1]. At this point, it is further indicated by pathogenesis changes in brain structure and cognitive function in aging [2]. To extend healthy lifespan of old people, prevent and delay attacks of AD, we consider using the strategy of anti-aging for brain. In addition, anti-oxidation and anti-inflammation are the common action mechanisms of neuroprotection and anti-aging agents [3,4,5]. Some evidences have reported that resveratrol, curcumin, metformin, and rapamycin with anti-aging effects have neuroprotective function and show significant effects on AD animal models [6,7,8,9,10,11,12]. Thus, a necessary connection exists between them and anti-aging substances may be developed to promising neuroprotective drugs. 

AD is a degenerative condition characterized by decline in memory and cognitive abilities because of neuronal dysfunction or death, and has tormented millions of people around the world [13,14]. The drugs treated AD have tacrine, rivastigmine, galantamine, donepezil and memantine. However, these drugs can only temporarily improve AD symptoms and do not cure the disease [13,14]. Thus, further discovery of novel and effective drugs based on different theories and bioassay systems remains an urgent necessity.

In our previous studies, we searched for anti-aging compounds from food and Chinese herb medicine with the replicative lifespan assays of K6001 yeast, *Saccharomyces cerevisiae*. We found that ganodermasides A-B, phloridzin, and nolinospiroside F can extend the lifespan of yeast via anti-oxidative stress and regulation of *Sir2*, *UTH1* expression [15,16,17]. In the present study, we turn our focus on marine products and report that mussel (*Mytilidae*), which was screened from over 70 sea foods, shows significant anti-aging effects. An active sterol fraction (SF) and four compounds were obtained from mussels, and studies on the action mechanism of these compounds suggests that mussel sterols exert anti-oxidative stress functions via *UTH1* and *SOD* and extend the replicative lifespan of yeast. Based on the connection between neuroprotection and anti-aging, neuroprotective experiments were performed on PC12 cells derived from rat pheochromocytoma cells. As expected, cholesterol (CHOL) and SF rescued the cells from injury induced by paraquat and showed nerve growth factor (NGF) mimic activity. Sterols from mussel show potential for application in AD treatment because of their anti-aging and neuroprotective functions.

## 2. Results and Discussion

### 2.1. SF, Sterols, and Their Anti-Aging Activities

**Figure 1:**
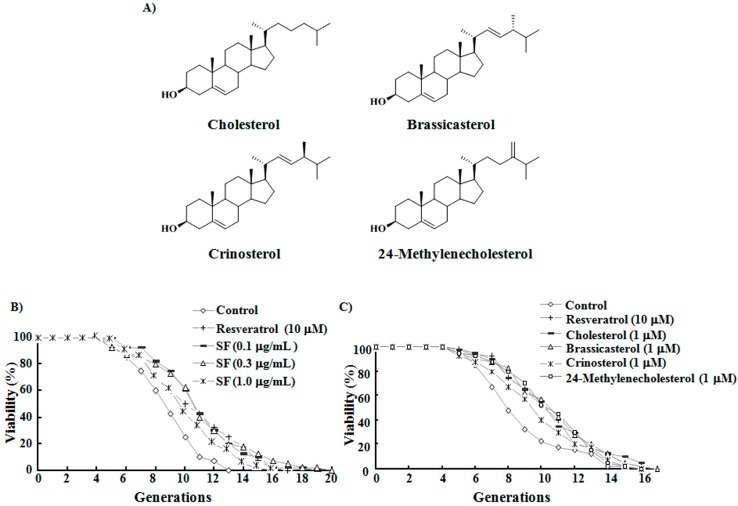
Anti-aging activities of sterol fraction (SF) and the four sterol compounds obtained from this fraction and their respective chemical structures. (**A**) Chemical structures of the four sterol compounds; (**B**) Effects of SF on the replicative lifespan of K6001 yeast. In the replicative lifespan assay, 4000 cells were spread on glucose agar plates. Daughter cells of 40 microcolonies in each plate were counted. The average lifespan of the K6001 control group was 8.0 ± 0.34 generations; resveratrol at 10 μM, 9.7 ± 0.53 **; SF at 0.1 μg/mL, 10.3 ± 0.46 ***; SF at 0.3 μg/mL, 10.2 ± 0.56 **; SF at 1 μg/mL, 9.2 ± 0.43 *; (**C**) Effects of the four sterols on the replicative lifespan of K6001. The average lifespan of the K6001 control group was 8.0 ± 0.43 generations; resveratrol at 10 μM, 9.7 ± 0.44 **; cholesterol (CHOL) at 1 µM, 9.9 ± 0.47 **; brassicasterol at 1 µM, 9.9 ± 0.47 **; crinosterol at 1 µM, 10.0 ± 0.46 **; 24-methylenecholesterol at 1 µM, 9.8 ± 0.43 **. * *p* < 0.05, ** *p* < 0.01, and *** *p* < 0.001 indicate statistically significant difference.

Mussel samples were separated under guidance of a bioassay system, and an active SF was obtained. This fraction was mainly composed of steroids (70%). SF was further purified by reversed-phase high performance liquid chromatography (HPLC) to yield four compounds, the structures of which were determined as cholesterol [18,19], brassicasterol [20,21], crinosterol [20,22], and 24-methylenecholesterol [23] (Figure 1A) based on their spectroscopic characteristics and comparison of spectroscopic data with those in the literature. Quantitative analysis showed that the percentages of CHOL, brassicasterol, crinosterol, and 24-methylenecholesterol in the SF were 27%, 19%, 12%, and 12%, respectively.

The replicative lifespan of K6001 after SF administration was measured. Results in Figure 1B show that treatment with 0.1, 0.3, or 1 μg/mL SF extends the replicative lifespan of yeast (*p* < 0.001, *p* < 0.01, and *p* < 0.05, respectively). The biological activities of the four sterol compounds at concentrations of 0.3, 1, 3, and 10 μM were also investigated (Figure S1). The four compounds similarly prolonged the lifespan of yeast at the optimized concentration of 1 μM as shown in Figure 1C. These results indicate that SF and the four compounds possess anti-aging effects in yeast.

### 2.2. CHOL Improved the Oxidative Resistance of Yeast

**Figure 2:**
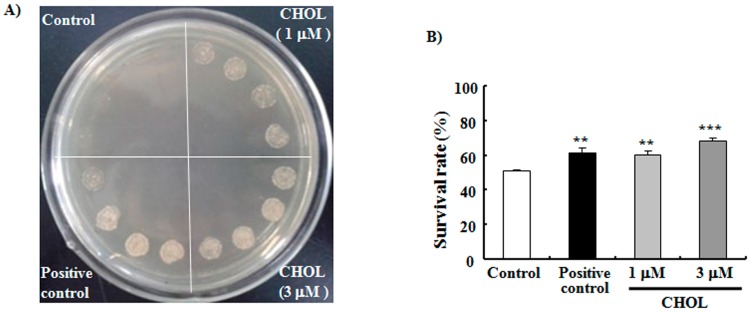
Effects of CHOL on the oxidative resistance of yeast. (**A**) BY4741 yeast was incubated for 12 h after treatment with 0, 1, or 3 μM CHOL. Cell culture solutions (5 μL) with the same OD were dropped onto plates containing 9 mM H_2_O_2_. The plates were incubated for 4 days at 28 °C and subsequently photographed. Positive control: 10 μM phloridzin; (**B**) BY4741 yeast cells were spread on glucose agar plates with or without 4 mM H_2_O_2_. The plates were then incubated for 2 days at 28 °C. Each experiment was conducted at least three times. Vertical bars represent the mean SEM of three assays. ** *p* < 0.01 and *** *p* < 0.001 indicates statistically significant difference.

Among the four compounds obtained, all of which have similar anti-aging effects at the optimum concentration of 1 µM, CHOL was the most abundant. As such, we investigated the action mechanism of CHOL. Oxidative stress is considered the main cause of aging in various organisms [24]. CHOL is thought to be an antioxidant and can be oxidized to oxysterols by enzymes of the cytochrome P450 family, reactive oxygen species (ROS) and light exposure [25,26]. Therefore, we firstly sought to determine whether this sterol could improve the oxidative resistance of yeast. Results in Figure 2A demonstrate that treatment with 1 or 3 μM CHOL significantly improves the survival of yeast under oxidative stress conditions caused by 9 mM H_2_O_2_. In another experiment, we measured the change of survival rate under oxidative stress conditions induced by 4 mM H_2_O_2_ to quantize observed improvement. Yeast cells were spread on agar plates with and without 4 mM H_2_O_2_, and survival rates were calculated as the ratio of colony units between the two plates. The survival rates of the control, positive control, 1 μM CHOL-treated, and 3 μM CHOL-treated groups were 51.0% ± 0.8%, 61.5% ± 2.9% (*p* < 0.01), 60.6% ± 2.1% (*p* < 0.01) and 68.4% ± 1.9% (*p* < 0.001), respectively (Figure 2B). Survival rates were significantly improved by CHOL, and these results indicate that CHOL presents anti-oxidative functions.

### 2.3. CHOL Decreased ROS and MDA Production

**Figure 3:**
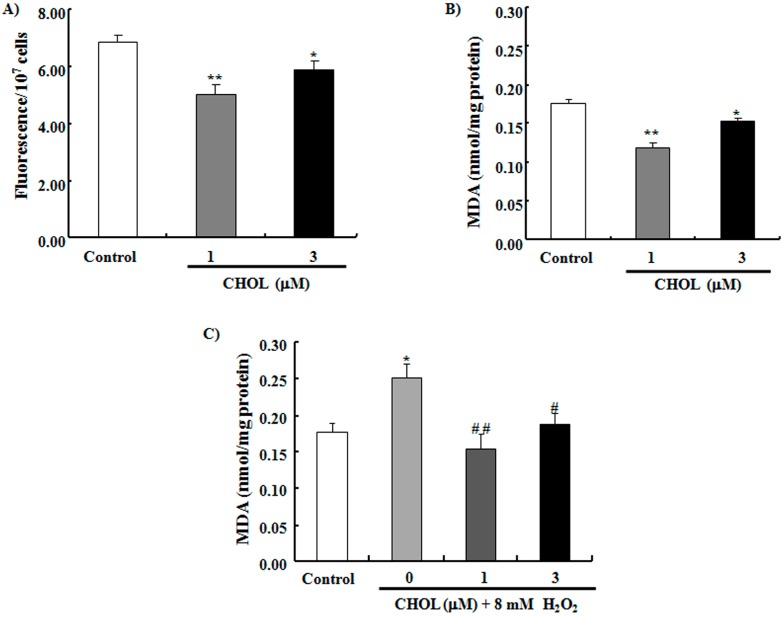
Effects of CHOL on reactive oxygen species (ROS) and malondialdehyde (MDA) production in yeast. (**A**) Effect of CHOL on ROS production. Fluorescence/(10^7^ cells) in the control group was 6.83 ± 0.28; CHOL at 1 μM, 5.03 ± 0.35; CHOL at 3 μM, 5.91 ± 0.30; (**B**) Effect of CHOL on endogenous MDA production of yeast. MDA production in the control group was 0.18 ± 0.005 nmol/mg protein, CHOL at 1 μM, 0.12 ± 0.006; CHOL at 3 μM, 0.15 ± 0.003; (**C**) Effect of CHOL on MDA production of yeast under oxidative stress condition. MDA production in the control group was 0.18 ± 0.01 nmol/mg protein; Control group treated with 8 mM H_2_O_2_, 0.25 ± 0.02; CHOL at 1 μM, 0.16 ± 0.02; CHOL at 3 μM, 0.19 ± 0.02. Each experiment was conducted at least three times. Vertical bars represent the mean SEM of three assays. * *p* < 0.05 and ** *p* < 0.01 indicate statistically significant difference from the corresponding control. ^#^ *p* < 0.05 and ^##^ *p* *<* 0.01 indicate statistically significant difference from the control treated with 8 mM H_2_O_2_.

Reactive oxygen species (ROS) are harmful metabolites produced by mitochondria even in normal conditions. These substances destroy the cytomembrane and biological macromolecules, such as proteins and nucleic acids, subsequently disturbing cell functions and causing aging and death [27]. We tested the ROS level of yeast after CHOL administration. Figure 3A shows that the ROS of yeast significantly decreased after treatment with 1 μM (*p* < 0.01) or 3 μM (*p* < 0.05) CHOL. ROS destroys the cell membrane by oxidizing unsaturated fatty acids and further produces harmful substances, such as malondialdehyde (MDA) [28]. MDA production can reflect the oxidation degree of lipids in organisms. As such, we examined whether or not CHOL can lower MDA production. Figure 3B dispalys that CHOL reduced endogenous MDA production of yeast (*p* < 0.01, *p* < 0.05). Furthermore, we investigated MDA production under oxidative stress condition by H_2_O_2_. Figure 3C shows that MDA production in yeast treated with 8 mM H_2_O_2_ significantly increased (*p* < 0.05) and decreased after treatment with 1 or 3 μM CHOL (*p* < 0.01, *p* < 0.05). These results suggest that CHOL decreases ROS and MDA production in yeast; this effect may exert an important function in the anti-aging process.

**Figure 4:**
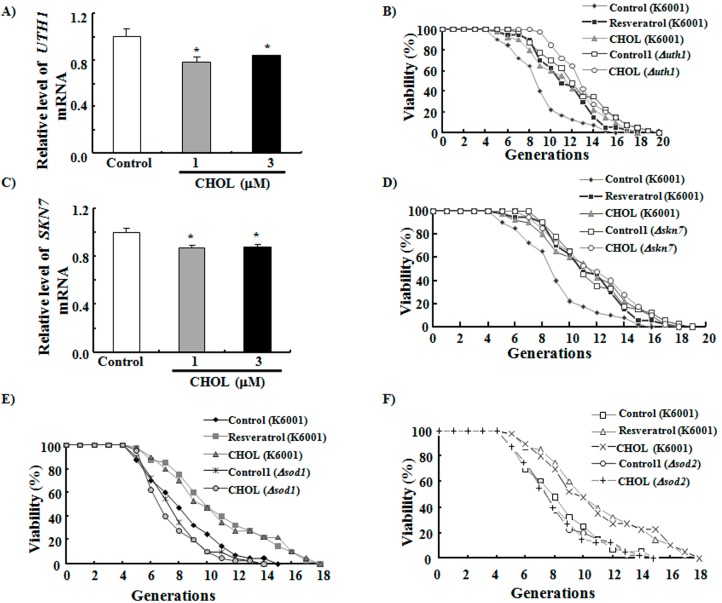
Effects of CHOL on *UTH1* and *SKN7* genes expression and the replicative lifespans of *uth1*, *skn7*, *sod1*, and *sod2* mutations. (**A**) Effects of CHOL on *UTH1* gene expression. The relative level of *UTH1* mRNA in control group is 1.01 ± 0.06; CHOL at 1 μM, 0.79 ± 0.04 *; CHOL at 3 μM, 0.84 ± 0.01 *; (**B**) Effect of CHOL on *uth1* mutation with a K6001 background. The average lifespan of the K6001 control group was 8.3 ± 0.44 generations; resveratrol at 10 μM, 10.7 ± 0.49 ***; CHOL at 1 µM, 10.7 ± 0.55 ***. The average lifespan of the *uth1* mutation control1 group was 11.7 ± 0.53 generations; CHOL at 1 µM, 12.4 ± 0.42; (**C**) Effects of CHOL on *SKN7* gene expression. The relative level of *SKN7* mRNA in control group is 1.00 ± 0.03; CHOL at 1 μM, 0.87 ± 0.02 *; CHOL at 3 μM, 0.88 ± 0.03 *; (**D**) Effect of CHOL on *skn7* mutation with a K6001 background. The average lifespan of the K6001 control group was 8.3 ± 0.44 generations; resveratrol at 10 μM, 10.7 ± 0.49 ***; CHOL at 1 µM, 10.7 ± 0.55 ***. The average lifespan of the *skn7* mutation control1 group was 11.0 ± 0.47 generations; CHOL at 1 µM, 11.2 ± 0.50; (**E**) Effect of CHOL on *sod1* mutation with a K6001 background. The average lifespan of the K6001 control group was 7.6 ± 0.43 generations; resveratrol at 10 μM, 10.0 ± 0.57 ***; CHOL at 1 µM, 9.9 ± 0.60 **. The average lifespan of the *sod1* mutation control1 group was 7.0 ± 0.35 generations; CHOL at 1 µM, 6.7 ± 0.35; (**F**) Effect of CHOL on *sod2* mutation with a K6001 background. The average lifespan of the K6001 control group was 7.6 ± 0.43 generations; resveratrol at 10 μM, 10.0 ± 0.57 ***; CHOL at 1 µM, 9.9 ± 0.60 **. The average lifespan of the *sod2* mutation control1 group was 7.3 ± 0.41 generations; CHOL at 1 µM, 7.3 ± 0.41. * *p* < 0.05, ** *p* < 0.01 and *** *p* < 0.001 indicate statistically significant difference.

**Figure 5:**
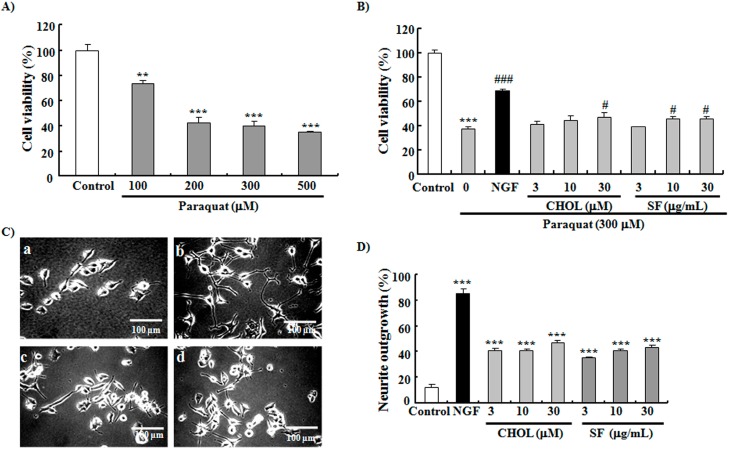
Neuroprotective effects of CHOL and SF. (**A**) The dose-dependent investigation of paraquat. The cell viability of the control group was 100.0% ± 4.4%; 100 μM paraquat, 73.4% ± 3.1%; 200 μM paraquat, 41.9% ± 4.6%; 300 μM paraquat, 39.6% ± 3.8%; 500 μM paraquat, 34.9% ± 0.4%; (**B**) Effects of SF and CHOL on survival viability of PC12 cells under oxidative stress induced by 300 μM paraquat. Control: 0.5% DMSO; NGF: positive control, 40 ng/mL. The cell viability in the control group was 100.0% ± 3.0%; the control group treated with 300 μM paraquat, 37.9% ± 1.7%; NGF, 69.5% ± 1.4%; CHOL at 3 μM, 41.5% ± 2.6%; CHOL at 10 μM, 44.7% ± 4.1%; CHOL at 30 μM, 46.8% ± 4.5%; SF at 3 μg/mL, 39.0% ± 0.4%; SF at 10 μg/mL, 45.9% ± 1.9%; SF at 30 μg/mL, 45.5% ± 2.4%; (**C**) Photomicrographs of PC12 cells after treatment with SF and CHOL: (a) Solvent control (0.5% DMSO), (b) NGF (40 ng/mL), (c) CHOL (30 μM), and (d) SF (30 μg/mL); (**D**) Percentage of neurite outgrowth of PC12 cells treated with 3, 10, or 30 μM CHOL or 3, 10, or 30 μg/mL SF. The percentage of neurite outgrowth of PC12 cells in the control group was 13% ± 1.8%; NGF, 85% ± 3.7%; SF at 3 μg/mL, 35% ± 1.2%; SF at 10 μg/mL, 41% ± 1.2%; SF at 30 μg/mL, 43% ± 1.2%; CHOL at 3 μM, 41% ± 2.0%; CHOL at 10 μM, 41% ± 1.2%; CHOL at 30 μM, 47% ± 2.1%. Each experiment was conducted at least three times. Vertical bars represent the mean SEM of three assays. ** *p* < 0.01, and *** *p* < 0.001 indicate statistically significant difference from the corresponding control. ^#^ *p* < 0.05 and ^###^ *p* < 0.001 indicate significant difference from the control treated with 300 μM paraquat.

### 2.4. Anti-Aging Effects of CHOL Diminished in Uth1, Skn7, Sod1, and Sod2 Mutations with a K6001 Background

Yeast cells with *UTH1* deletion exhibit increased lifespans during starvation stress and show altered sensitivities to oxidative damage [29]. However, yeast Skn7, a transcription factor, senses stress and activates oxidative stress response genes [30]. These two genes performed important functions in the anti-aging activities of many active compounds in our previous studies [15,17]. Here, the effects of CHOL on *UTH1* and *SKN7* genes expression of K6001 yeast and the replicative lifespans of *uth1* and *skn7* mutations with a K6001 background were investigated. We discovered that CHOL significantly inhibited the genes expression of *UTH1* and *SKN7* (Figure 4A,C), the anti-aging effects of CHOL were diminished under the two mutations (Figure 4B,D). We thus infer that one of the mechanisms of the anti-aging effects of CHOL on K6001 involves improvement of oxidative stress responses by regulating *UTH1* and *SKN7*. Next, *sod1* and *sod2* mutations were employed to check whether *SOD* genes take important role in anti-aging effects of CHOL and SF. As expected, CHOL did not extend the lifespans of *sod1* and *sod2* mutations (Figure 4E,F). These results suggest that *UTH1* and *SOD* genes are involved in the anti-aging effects of sterol compounds.

### 2.5. SF and CHOL Exerted Neuroprotective Effects on PC12 Cells

Anti-oxidative stress is one of the most important mechanisms of neuroprotective function [3,4,5]. We hypothesized that sterols from mussels may have neuroprotective functions. To prove this hypothesis, we used paraquat to induce oxidative stress conditions in PC12 cells and investigate the neuroprotective effects of SF and CHOL. As expected, paraquat dose-dependently reduced cell viability, as seen in Figure 5A. SF at concentrations of 10 and 30 μg/mL and CHOL at 30 μM improved reductions in cell viability induced by 300 μM paraquat, as seen in Figure 5B (*p* < 0.001, *p* < 0.05, *p* < 0.05, and *p* < 0.05, respectively). In general, compounds with NGF mimic activities exert neuroprotective functions because of the broad favorable effects of NGF on nerve cells [31]. In another experiment, NGF mimic effects of SF and CHOL were observed on PC12 cells. Figure 5C shows morphological changes in the PC12 cells after treatment with SF and CHOL. Figure 5D shows that the percentages of neurite outgrowth of PC12 cells increased after treatment with 3, 10, or 30 μg/mL SF (*p* < 0.001, *p* < 0.001, and *p* < 0.001, respectively) or 3, 10, or 30 μM CHOL (*p* < 0.001, *p* < 0.001, and *p* < 0.001, respectively). These results confirm that CHOL and SF have neuroprotective functions. Since paraquat is one kind of toxicant with strong toxicity. It leads oxidative stress by targeting mitochondria to produce reactive oxygen species and depolarization of mitochondria membrane. This process is irreversible. It is possible to lead that the effects of cholesterol and SF on cell viability in paraquat-treated PC12 cells was quite small.

## 3. Experimental Section

### 3.1. Extraction and Isolation

Mussel samples were purchased from Donghe Market of Zhoushan, Zhejiang Province, China, in 2012. Approximately 179.0 g (dry weight) of the sample was mashed and extracted with methanol for 72 h with shaking at room temperature. The extraction solution was partitioned between 80% methanol-water and *n-*hexane (volume ratio 1:1). The *n-*hexane layer was concentrated by rotary evaporation and decompressed to obtain 4.0 g of crude sample. This sample of 2.0 g was later separated by silica gel (200–300 mesh, Yantai Chemical Industry Research Institute, Yantai, China) with *n-*hexane/EtOAc (99:1, 98:2, 97:3, 96:4, 90:10, 0:100) and 100% MeOH. The combined active fractions were eluted with *n-*hexane/EtOAc (90:10) and purified by ODS with MeOH/H_2_O (90:10, 93:7, 95:5, and 100:0). One SF of 147.0 mg was acquired, of which 16.7 mg was purified by reversed-phase HPLC (Develosil ODS-HG-5 (φ 10/250 mm), Nomura Chemical, flow rate: 3 mL/min, 90% MeOH/H_2_O) to obtain CHOL (4.5 mg, *t*_R_ = 160.9 min), brassicasterol (3.2 mg, *t*_R_ = 146.7 min), crinosterol (2.0 mg, *t*_R_ = 136.9 min), and 24-methylenecholesterol (2.0 mg, *t*_R_ = 124.0 min).

Cholesterol: a colorless powder, ${\left[\alpha \right]}_{D}^{19}$-34 (*c* 0.42, CHCl_3_). High-resolution ESI-TOF-MS *m/z* 409.345, calcd. for C_27_H_46_ONa (M + Na)^+^ 409.344. ^1^H NMR (500 MHz, CDCl_3_): δ = 0.68 (s, 3H), 0.86 (d, 3H, *J* = 2.0 Hz), 0.87 (d, 3H, *J* = 2.0 Hz), 0.92 (d, 3H, *J* = 6.5 Hz), 1.01 (s, 3H), 3.52 (m, 1H), 5.36 (m, 1H). ^13^C NMR (125 MHz, CDCl_3_): *δ* = 12.0, 18.9, 19.5, 21.2, 22.7, 23.0, 24.0, 24.5, 28.1, 28.4, 31.8, 32.1, 32.1, 35.9, 36.3, 36.7, 37.4, 39.7, 39.9, 42.5, 42.5, 50.3, 56.3, 56.9, 72.0, 122.2, 141.1. The spectra of ^1^H, ^13^C NMR were displayed in Figure S2A,B.

Brassicasterol: a colorless powder, ${\left[\alpha \right]}_{D}^{25}$-59 (*c* 0.28, CHCl_3_). High-resolution ESI-TOF-MS *m*/*z* 421.345, calcd. for C_28_H_46_ONa (M + Na)^+^ 421.344. ^1^H NMR (500 MHz, CDCl_3_): δ = 0.69 (s, 3H), 0.82 (d, 3H, *J* = 6.5 Hz), 0.83 (d, 3H, *J* = 6.5 Hz), 0.91 (d, 3H, *J* = 6.8 Hz), 1.01 (s, 3H), 1.01 (d, 3H, *J* = 6.5 Hz), 3.52 (m, 1H), 5.18 (m, 2H), 5.34 (m, 1H). ^13^C NMR (125 MHz, CDCl_3_): δ = 12.2, 17.8, 19.6, 19.8, 20.1, 21.1, 21.2, 24.4, 28.7, 29.9, 31.8, 32.1, 33.3, 36.7, 37.4, 39.8, 40.3, 42.4, 42.5, 43.0, 50.3, 56.2, 57.0, 72.0, 121.9, 131.9, 136.0, 140.9. The spectra of ^1^H, ^13^C NMR were given in Figure S3A,B.

Crinosterol: a colorless powder, ${\left[\alpha \right]}_{D}^{24}$-44 (*c* 0.17, CHCl_3_). High-resolution ESI-TOF-MS *m*/*z* 421.345, calcd. for C_28_H_46_ONa (M + Na)^+^ 421.344. ^1^H NMR (500 MHz, CDCl_3_): δ = 0.69 (s, 3H), 0.82 (d, 3H, *J* = 6.8 Hz), 0.84 (d, 3H, *J* = 6.8 Hz), 0.91 (d, 3H, *J* = 6.8 Hz), 1.00 (d, 3H, *J* = 6.9 Hz), 1.01 (s, 3H), 3.52 (m, 1H), 5.16 (m, 2H), 5.35 (m, 1H). ^13^C NMR (125 MHz, CDCl_3_): δ = 12.2, 18.2, 19.6, 19.8, 20.3, 21.2, 21.2, 24.5, 29.0, 29.9, 31.9, 32.1, 33.4, 36.7, 37.4, 39.9, 40.4, 42.4, 42.5, 43.2, 50.3, 56.1, 57.1, 72.0, 121.9, 132.0, 136.2, 141.0. The spectra of ^1^H, ^13^C NMR were shown in Figure S4A,B.

24-Methylenecholesterol: a colorless powder, ${\left[\alpha \right]}_{D}^{25}$-35 (*c* 0.28, CHCl_3_). High-resolution ESI-TOF-MS *m*/*z* 421.344, calcd. for C_28_H_46_ONa (M + Na)^+^ 421.344. ^1^H NMR (500 MHz, CDCl_3_): δ = 0.68 (s, 3H), 0.95 (d, 3H, *J* = 6.6 Hz), 1.01 (s, 3H), 1.02 (d, 3H, *J* = 6.9 Hz), 1.03 (d, 3H, *J* = 6.9 Hz), 3.53 (m, 1H), 4.66 (s, 1H), 4.71 (s, 1H), 5.35 (m, 1H). ^13^C NMR (125 MHz, CDCl_3_): δ = 12.0, 18.9, 19.6, 21.3, 22.0, 22.2, 24.5, 28.4, 29.9, 31.2, 31.9, 32.1, 34.0, 34.9, 35.9, 36.7, 37.4, 40.0, 42.5, 42.5, 50.3, 56.2, 56.9, 72.0, 106.1, 121.9, 140.9, 157.1. The spectra of ^1^H, ^13^C NMR were displayed in Figure S5A,B.

The chromatograms from the LC-MS purification and HRMS spectra of the four compounds were displayed in Figure S6.

### 3.2. Chemical Analysis

Nuclear magnetic resonance spectra were recorded on a Bruker AV III-500 spectrometer (Bruker, Billerica, MA, USA). Chemical shifts are shown in terms of δ (ppm), and signals are presented as singlet (s), doublet (d), and multiplet (m). Chemical shifts in δ (ppm) were referenced to the solvent peak of δ_H_ 7.26 and δ_C_ 77.0 for CDCl_3_. High-resolution ESI-TOF-MS analysis was performed on an Agilent Technologies 6224A accurate mass TOF LC/MS system (Santa Clara, CA, USA). All of the solvents used were of analytical reagent grade.

### 3.3. Strains, Culture Media, and Lifespan Assay

In the present study, wild-type BY4741, K6001 with a W303 background, and *uth1*, *skn7*, *sod1*, and *sod2* mutations with a K6001 background were used. The preparation of SF and four compounds was given in the extraction and isolation of experimental section. Samples were separated under the guidance of yeast lifespan assays as described in a previous study [15]. Briefly, K6001 cells were inoculated in 5 mL of yeast-peptone-galactose medium and cultured for 48 h in a shaker at 28 °C and 160 rpm. The cultured broth was then centrifugated, and the yeast cells obtained were washed three times and diluted with phosphate buffer solution (PBS). Approximately 4000 yeast cells, counted using a hemocytometer, were coated on glucose agar plates containing different sample concentrations. The plates were incubated at 28 °C for 2 days, and daughter cells from 40 random microcolonies were counted in each plate. Each experiment was repeated three times.

### 3.4. Anti-Oxidative Stress Assay

Wild-type BY4741 yeast was inoculated in 5 mL of yeast-peptone-d-glucose (YPD) medium and cultured at 28 °C and 160 rpm in a shaker for 2 days. The yeast cells were treated with 25 mL of YPD medium at 0.1 OD_600_ and incubated for 12 h with 0, 1, or 3 μM CHOL and 10 µM phloridzin. Then, 5 μL of culture solution with the same number of cells in each group were dropped on to glucose agar plates containing 9 mM H_2_O_2_ and incubated at 28 °C for 4 days. Yeast growth on the plates was observed and photographed. Phloridzin was used as a positive control. Cell viability assays were performed to accurately measure the anti-oxidative abilities of CHOL. After incubation of the yeast with different concentrations of CHOL, approximately 200 cells were spread on glucose agar plates with or without 4 mM H_2_O_2_. These plates were incubated for 2 days at 28 °C, and the survival rates of the sample groups were compared with that of the control group.

### 3.5. ROS and MDA Assays

The ROS and MDA assay procedures have been described in a previous study [17] and are modified in the present study. K6001 yeast cells were cultured as described in the experiment above and incubated with CHOL for 23 h. Changes in intracellular ROS levels of the yeast were determined using an ROS assay kit (Beyotime, Jiangsu, China) and a fluorescent plate reader (Spectra Max M2, Molecular Devices, San Francisco, CA, USA). A total of 1 mL of cultured broth was obtained, treated with 10 µM DCFH-DA at 28 °C in the dark, and then shaken by vortexing at 160 rpm at 15 min intervals for 1 h. The yeast cells were subsequently washed with PBS, and their DCF fluorescence was measured by a fluorescent plate reader at excitation and emission wavelengths of 488 and 525 nm, respectively. Cell numbers were calculated by a hemocytometer and the DCF fluorescence produced by the same number of cells was compared.

During MDA assay, cells were incubated with CHOL for 23 h, treated with 8 mM H_2_O_2_ to induce oxidative stress or not, and then incubated sequentially for 1 h. All of the cells were collected and washed with PBS three times. Cells were resuspended in 500 μL of PBS and disintegrated by ultrasonication (1 min/time, 5 times). The cell lysate was centrifuged at 12,000 rpm at 4 °C for 15 min, and the MDA in the supernatant was measured using a kit according to the manufacturer’s instructions (MDA assay kit, Nanjing Jiancheng Bioengineering Institute, Nanjing, China).

### 3.6. RT-PCR Assay

RT-PCR was performed as previously described [17]. K6001 yeast cells were cultured in yeast-peptone-galactose medium and incubated with CHOL at 0, 1, and 3 μM for 12 h. Then RNA was extracted from the yeast cells by the hot-phenol method. RNA cleanup was done with an RNApure Tissue Kit (Beijing Cowin Biotech Company, Beijing, China). Reverse transcription was performed using a HiFi-MMLV cDNA kit (Beijing Cowin Biotech Company, Beijing, China), 5 μg of total RNA. Real-time PCR (RT-PCR) was done by CFX-Touch (Bio-rad, Hercules, CA, USA) and SYBR Premix EX Taq (Takara, Ohtsu, Japan). The thermal cycling parameters were as follows: 40 cycles, 95 °C for 15 s, 55.4 °C for 15 s, and 68 °C for 20 s. Primers used were as follows: for *UTH1*, sense 5'-CGC CTC TTC TTC CTC CTC TT-3' and antisense 5'-ACC ATC GGA AGG TTG TTC AG-3'; for *SKN7*, sense 5'-AGT TGT CAG CGA CGG TCT TT-3' and antisense 5'-GCT GTG GCA CCA TCT AGG TT-3'; for TUB1, sense 5'-CCA AGG GCT ATT TAC GTG GA-3' and antisense 5'-GGT GTA ATG GCC TCT TGC AT-3'. The amounts of *UTH1* and *SKN7* mRNA were normalized to that of TUB1.

### 3.7. Bioassay of NGF Mimic Activity and Neuroprotection Assay on PC12 Cells

Bioassay of NGF mimic activity was performed as described in our previous study [32]. A total of 20,000 cells were seeded in each well of a 24-well microplate and cultured in 5% CO_2_ at 37 °C. After 24 h, the medium was replaced with 1 mL of serum-free Dulbecco’s modified eagle medium (DMEM) containing 3, 10, or 30 μg/mL SF; 3, 10, or 30 μM CHOL; or DMSO (0.5%). NGF was used as a positive control. Morphological changes in the PC12 cells were observed under a phase-contrast microscope after incubation for 48 h. Approximately 100 cells were observed from a random area. A cell with neurite outgrowth longer than the diameter of the cell body was identified as a positive cell. 

In the neuroprotection assay, we used 100, 200, 300, and 500 μM paraquat to induce oxidative stress to determine the optimum dose that decreases 40% of the survival rate. PC12 cells were pretreated with NGF; 3, 10, or 30 μM CHOL; or 3, 10, or 30 μg/mL SF for 2 h. Subsequent treatment with 300 μM paraquat was performed, and co-incubation proceeded for 48 h. Afterward, the medium was replaced with 500 μL of serum-free DMEM containing 200 μg/mL 3-(4,5-dimethylthiazol-2-yl)-2,5-diphenyltetrazolium bromide. Cells were then cultured at 37 °C for another 2 h. Finally, the medium was completely removed from the plate and 200 μL of DMSO was added to each well to solubilize the formazan crystals that had formed. The resultant formazan was detected at 570 nm using a plate reader (Bio-Tek instruments Inc., Winooski, VT, USA).

### 3.8. Statistical Analysis

Significant differences among groups in all experiments were determined by ANOVA followed by two-tailed multiple *t*-tests with Bonferroni correction using SPSS Biostatistics software [33]. A *p*-value less than 0.05 was considered statistically significant.

## 4. Conclusions

In this paper, four anti-aging compounds were isolated from mussel samples. The structures of these compounds were determined, and the compounds were used in replicative lifespan assays of K6001 yeast. All of the compounds were sterols, and their sterol skeletons performed important functions in their anti-aging effects. Action mechanism studies demonstrated that the anti-aging effects of the sterols depended on their anti-oxidative ability, including reduction of the ROS and MDA levels of yeast. We also found that the *UTH1* and *SOD* genes are involved in the anti-aging effects of CHOL. Anti-oxidative stress is an important mechanism of neuroprotective function. As such, we confirmed the neuroprotective functions and NGF mimic activity on PC12 cells. In future studies, we will perform animal experiments to determine whether these isolated compounds and SF have therapeutic effects on AD.

## Supplementary Materials

Supplementary figures can be found at http://www.mdpi.com/1422-0067/15/12/21660/s1.

## Abbreviations

AD: Alzheimer’s disease
CHOL: cholesterol
DMEM: Dulbecco’s modified eagle medium
HPLC: high performance liquid chromatography
MDA: malonaldehyde
NGF: nerve growth factor
PBS: phosphate buffer solution
ROS: reactive oxidative species
SF: sterol fraction
YPD: yeast peptone d-glucose
